# Nutritional Status and Dietary Challenges in Patients with Systemic Sclerosis: A Comprehensive Review

**DOI:** 10.3390/nu17193144

**Published:** 2025-10-01

**Authors:** Eleni C. Pardali, Arriana Gkouvi, Maria G. Grammatikopoulou, Alexandros Mitropoulos, Christos Cholevas, Dimitrios Poulimeneas, Markos Klonizakis

**Affiliations:** 1 Immunonutrition Unit, Department of Rheumatology and Clinical Immunology, Faculty of Medicine, School of Health Sciences, University of Thessaly, Biopolis, GR-41223 Larissa, Greece; elpardali@uth.gr (E.C.P.); arriana_gk@yahoo.gr (A.G.); 2 Lifestyle, Exercise and Nutrition Improvement (LENI) Research Group, Department of Nursing and Midwifery, Sheffield Hallam University, Collegiate Hall, Collegiate Crescent Rd, Sheffield S10 2BP, UK; 3 Laboratory of Pharmaceutical Technology, Division of Pharmaceutical Technology, School of Pharmacy, Faculty of Health Sciences, Aristotle University of Thessaloniki, GR-54124 Thessaloniki, Greece; 4 Department of Nutritional Science and Dietetics, School of Health Sciences, University of the Peloponnese, GR-24100 Kalamata, Greece; dpoul@hua.gr; 5 Department of Nutrition and Dietetics, School of Health Sciences and Education, Harokopio University, GR-17778 Athens, Greece

**Keywords:** malnutrition, sarcopenia, malabsorption syndrome, gastrointestinal tract, nutrition, diet, dietary supplements, frailty, medical nutrition therapy

## Abstract

The gastrointestinal (GI) tract is seriously affected by systemic sclerosis (SSc), due to fibrosis and persistent inflammation. Patients with GI involvement frequently exhibit poor nutritional status, which affects disease burden and quality of life. The aim of the present review was to discuss all nutritional issues in SSc and serve as a primer for the nutritional assessment of patients with scleroderma. Patients with SSc suffer from GI impairments that affect the oral cavity, esophagus, stomach, and small and large intestines. Symptomatology includes microstomia, xerostomia, dysphagia, reflux, esophageal dysmotility, small intestinal bacterial overgrowth (SIBO), and fecal incontinence, among others, which may contribute to inadequate food intake. As a result, patients often suffer from malnutrition, sarcopenia, and frailty, while presenting with micronutrient deficiencies that impact disease outcomes and worsen their condition. This aggravated nutritional status is related to greater disease severity, organ involvement, reduced physical function, and increased length of hospitalization and mortality. GI involvement is well-documented within the SSc population, yet routine nutritional assessments are lacking in the hospital setting. Currently, there is a lack of specific recommendations from scientific societies regarding the nutritional care of patients with SSc. Given the high risk of nutritional impairments in this population, systematic assessments should be undertaken, and novel tools tailored to their unique needs should be developed and implemented.

## 1. Introduction

Systemic sclerosis (SSc) is a chronic autoimmune connective tissue disease characterized by microvascular dysfunction and progressive fibrosis of the skin and internal organs [1]. It affects approximately 1.48 million people worldwide, with a higher incidence in women [2]. The chronic inflammatory and fibrotic processes involving the gastrointestinal (GI) tract, lungs, and skin in SSc often contribute to the deterioration of nutritional status [1] that occurs in both subtypes of SSc, diffuse (dSSc) and limited cutaneous (lSSc) [3].

After the skin, the GI tract is the second most frequently affected system, with GI involvement reported in over 90% of patients [4]. Manifestations can affect the entire GI tract, leading to impaired motility, digestion, absorption, and excretion [5]. GI manifestations of SSc often appear early in the disease course, tend to be progressive, and represent a major cause of morbidity and mortality [5,6]. Several mechanisms contribute to the deterioration of nutritional status among patients with scleroderma. Chronic inflammation and fibrosis lead to dysmotility and malabsorption [7]. The presence of oral and oropharyngeal manifestations, such as microstomia and dental problems, has been shown to impede food intake by inducing mechanical difficulties during mastication and swallowing [8]. Smooth muscle atrophy and vasculopathy are the major contributors to impaired motility [9,10], as they lead to denervation of the smooth muscle layer of the GI tract [11].

Nutritional impairments associated with SSc have significant clinical consequences. Malnutrition, sarcopenia, and frailty are linked to reduced muscle strength [12,13], impaired wound healing, greater susceptibility to infections [14], and diminished quality of life (QoL) [15]. These conditions may also worsen disease burden by exacerbating fatigue and weakness, limiting physical function, and increasing vulnerability to organ complications [16,17]. Nutritional deficiencies—such as inadequate levels of vitamins, minerals, and trace elements—may further impair immune function and tissue repair [18]. Evidence suggests that compromised nutritional status is associated with poorer treatment tolerance and greater morbidity and mortality in SSc, underscoring the importance of prompt recognition and targeted nutritional intervention [19]. Moreover, malnutrition in SSc not only worsens prognosis but may also compromise pharmacotherapy options. GI dysmotility can lead to impaired oral drug absorption and affect pharmacokinetics [20]. For instance, mycophenolate mofetil (MMF) consists of an established treatment option in SSc, showing large variability in drug exposure, with up to eight-fold differences between patients [21]. Lower levels of its active metabolite have been linked to intestinal inflammation and proton pump inhibitor (PPI) use, suggesting that malnutrition can affect MMF efficacy [21].

Despite the numerous nutritional issues accompanying SSc, nutritional assessment is still not routinely integrated into patient care. This lack of systematic evaluation results in the frequent underdiagnosis of malnutrition and, consequently, a poor nutritional status. These conditions often remain undetected until they reach an advanced stage, at which targeted interventions may prove less effective. The present narrative review aimed to synthesize the existing evidence on the nutritional status of patients with SSc and to showcase the importance of incorporating systematic nutritional screening into routine clinical practice.

## 2. The GI Tract

Table 1 details the clinical manifestation of SSc in the GI tract. Oral cavity involvement occurs in approximately 80% of patients with SSc [22]. Clinical manifestations may include microstomia, xerostomia, dental caries, periodontal disease, as well as bone or joint involvement [22,23]. In some rare cases, dystrophic calcinosis further aggravates oral dysfunction [22]. These clinical features have an adverse impact on mastication, speech, and overall oral function, thereby reducing QoL [22]. Furthermore, coexisting Sjögren’s syndrome (SS)—present in about 20% of individuals with SSc—can exacerbate salivary gland dysfunction, compounding xerostomia and further increasing the risk of oral disease [24]. Additionally, esophageal reflux, a concomitant symptom of SSc, raises oral cavity acidity and contributes to the growth of periodontal pathogens and dental wear [25]. Dietary strategies for individuals with chewing and swallowing difficulties focus on modifying food texture [26]. Increasing food’s cohesion, smoothness, or moisture minimizes the risk of aspiration and promotes safer consumption [26]. The International Dysphagia Diet Standardisation Initiative (IDDSI) establishes a globally standardized framework for classifying texture-modified foods and thickened liquids, ensuring consistent communication among clinicians, caregivers, and patients in the management of oropharyngeal dysphagia diets [27].

The esophagus is an organ primarily affected in all patients with SSc, presenting motor disorders [28]. A common problem involves dysmotility disorder, characterized by decreased amplitude of esophageal peristalsis and decreased lower esophageal sphincter pressure. Other symptoms include dysphagia [29], regurgitation, heartburn, and gastroesophageal reflux disease (GERD) [30,31]. Typically, clinicians initiate the evaluation of dysphagia with upper endoscopy, a procedure that can reveal GERD, structural lesions, and various types of esophagitis [32]. Dysphagia affects more than half of patients with SSc, with the reported prevalence reaching up to 76% [29], while increasing disease burden and reducing QoL [33]. The presence of esophageal dysmotility is determined using esophageal manometry, revealing diminished or absent peristalsis in the distal two-thirds of the esophagus and a hypotensive lower esophageal sphincter [32]. Although the esophagus does not play a direct role in the digestion process, it may be implicated in impaired digestion and/or absorption of nutrients, due to the decreased amount of food that reaches the stomach [34]. Dysphagia and reflux often lead to the avoidance of specific foods, which, in turn, leads to restricted diets that may lack protein, fiber, or micronutrients, while chronic PPI use for GERD can contribute to vitamin B12, Magnesium, and other micronutrient deficiencies [35,36,37]. Common dietary guidance for GERD includes consuming small, frequent meals, avoiding trigger foods such as fried and acidic items, and refraining from eating within 2–3 h before bedtime [38,39].

Stomach involvement is a common occurrence in patients with SSc, with a prevalence of up to 50% [40]. The manifestations of this condition include gastritis, gastroparesis, gastric ulcers, and gastric antral vascular ectasia (GAVE) [41,42,43], with associated symptoms being abdominal pain or distension, bloating, early satiety, and postprandial nausea [44]. The presence of GAVE has been related to worse health-related QoL [45], and is often linked to Iron deficiency anemia and increased gastric bleeding [46]. For gastroparesis, dietary recommendations focus on symptom relief by choosing soft, low-fat, and low-fiber foods and liquids, eating smaller, more frequent meals, and avoiding “problematic” foods like those with high-fiber content, fried foods, and raw vegetables [47]. In the management of ulcers, gastritis, and GAVE, patients are advised to abstain from spicy, acidic, and fried foods, chocolate, caffeine, and alcohol [48].

The small intestine is the second most commonly affected GI organ in SSc, after the esophagus [49,50]. Intestinal dysmotility occurs in 40–88% of patients, with small intestinal hypomotility being the primary abnormality [49]. This slowing of intestinal movement can cause pseudo-obstruction and often leads to small intestinal bacterial overgrowth (SIBO) [51,52]. SIBO is particularly common in SSc, with stagnant intestinal contents creating an environment where bacteria proliferate [51,52]. Patients with SSc experiencing SIBO frequently demonstrate notable weight loss over a six-month period and tend to report a greater burden of GI symptoms [51,52]. Beyond its symptomatic footprint, SIBO can cause malabsorption, further compromising nutritional status [53]. In SSc, shifts in the gut microbiota, causing dysbiosis, have been observed that mirror GI involvement [54,55]. These alterations have even been proposed as potential biomarkers of disease activity [54]. In rare cases, intestinal changes may also give rise to pneumatosis cystoides intestinalis (PCI) [56], while cases of celiac disease have also been observed [57]. Management of diarrhea may be supported by the inclusion of soluble fiber-rich foods such as bananas, applesauce, and white rice, along with easily digestible protein sources like meat, poultry, fish, and eggs [58]. High-fiber foods exceeding 2 g of fiber per serving should be avoided [58]. Additionally, probiotics have shown potential benefits, particularly in the case of SIBO [38]. A meta-analysis also revealed that most patients with SIBO showed improvements following antibiotic intake for 10–14 days with ciprofloxacin, amoxicillin, rifaximin, metronidazole, trimethoprim–sulfamethoxazole, or doxycycline [59]. In SSc, rifaximin and the combination of norfloxacin with metronidazole have also been evaluated, showing SIBO eradication rates of 73% at 1 month and 52% at 11 months post-treatment initiation, respectively [52,60,61].

Colon hypomotility is one of the most common complications, reported in 20–50% of patients with SSc [62]. It is characterized by colonic dilatation with loss of the normal haustral pattern and markedly reduced or absent contractile activity. Clinically, patients may experience either diarrhea or constipation [63]. Diarrhea is typically associated with SIBO, lymphatic fibrosis impairing drainage, and reduced intestinal permeability, all of which contribute to malabsorption [63,64,65,66]. In contrast, constipation and fecal incontinence result from decreased colonic compliance and delayed transit [63,67]. Fecal incontinence that may be present in most patients with SSc may also result from anorectal dysfunction. This condition, prevalent among these patients, is characterized by the presence of fecal leakage and urgency, thereby affecting QoL, as well as physical and mental well-being [68,69]. The internal anal sphincter, a smooth muscle analogous to the internal esophageal sphincter, is more commonly affected in SSc [70]. For decreased motility, dietary modifications include increasing dietary fiber intake, ensuring adequate hydration through increased fluid consumption, and engaging in regular exercise [38,39,71].

## 3. Dietary Intake of Patients with SSc

GI involvement has a marked impact on meal patterns and frequency [72]. In particular, patients with GERD or early satiety often avoid nighttime eating and reduce their daily meal number, frequently limiting intake to one or two meals [72]. Similarly, decreased appetite has been associated with substantially reduced meal frequency [72]. Rheumatologists also report that many of their patients modify their diet, with 71% indicating food avoidance or adherence to specific dietary regimens to alleviate symptoms [73].

Despite the importance of nutrition, patients with SSc underconsume fruits, vegetables and fiber [7]. Those affected by GERD tend to increase fat intake, whereas individuals with dysphagia more often rely on sweets, preserves, and snacks [72]. Frequent alcohol consumption has been associated with greater relative fat mass [72], while an inverse correlation has been observed between liver transaminase levels and the intake of nuts and seeds [72]. With regards to micronutrient intake, inadequate consumption of vitamin D, folate, Calcium, and Magnesium have been reported among patients, with insufficient Iron intake being particularly notable among women [74]. Research indicates that most patients tend to follow a Westernized diet high in sodium but frequently suboptimal with regard to energy intake [72]. Even when residing in Mediterranean countries [75], the majority of patients tend to adopt the Mediterranean diet (MedDiet) in a suboptimal manner. On the other hand, poorer adherence to the MedDiet was related to depressive mood, greater time missed at work, and severer Raynaud’s phenomenon perception and digital ulcers [75]. Alongside these patterns, many patients adopt targeted dietary approaches such as the low fermentable oligosaccharides, disaccharides, monosaccharides, and polyols (FODMAP) diet or swallowing-related strategies, including avoiding dry foods or preferring a soft diet [73].

Most (71%) patients with SSc report willingness to visit a dietitian [76], in particular during times when their symptoms aggravate or change. Their most important concerns regarding nutrition involve scleroderma symptom management and advice with regards to unintentional weight loss.

## 4. Malnutrition in SSc

Malnutrition is increasingly recognized as a significant complication in patients with SSc, with various clinical, nutritional, and prognostic implications. According to the recommendations of a North American expert panel [77], physicians should screen all patients with SSc for malnutrition; however, this does not appear to be the case in everyday clinical practice. The prevalence of malnutrition varies according to the assessment tool applied. Malnutrition and unintentional weight loss are apparent at high rates in SSc and are associated with pulmonary hypertension, heart failure, and albumin levels, as well as the extent of skin fibrosis, physical activity levels, and small intestine involvement [72,78,79,80]. Furthermore, disease activity index (DAI) and disease severity scale (DSS) are significantly higher in malnourished compared to well-nourished patients [81].

Using the Malnutrition Universal Screening Tool (MUST), the reported risk of moderate malnutrition ranges from 6% [82,83] to 37% [84], while severe malnutrition ranges from 3.64% [1] to 38.3% [85] (Table 2).

According to the European Society of Clinical Nutrition and Metabolism (ESPEN) criteria, the prevalence of malnutrition ranges between 8.8% [81] to 37.8% [16] in scleroderma and reaches up to 63.8% in women, post-menopause [79]. When applying the Global Leadership Initiative on Malnutrition (GLIM) criteria, 11.5% [100] to 50% [105] of patients with SSc demonstrate moderate malnutrition, whereas 8.7% [100] to 34.5% exhibit severe malnutrition. On the other hand, when the Controlling Nutritional Status (CONUT) score was applied, it failed to detect a high malnutrition prevalence [93]. In parallel, lower serum albumin levels are a consistent finding in patients with malnutrition [72,82], which is also persistent and apparent even when malnutrition is simply defined by body mass index (BMI) status (i.e., underweight) [88]. The wide range of malnutrition prevalence reported in the literature reflects the use of different screening and assessment tools, which do not fully overlap in the components of nutritional status they assess (Table 3). Each tool has inherent limitations when applied to patients with an SSc diagnosis and is further subject to potential investigator bias. In addition, pharmacotherapy, disease severity and progression, and variability in GI phenotypes are pivotal when determining nutritional risk.

Evidence from longitudinal studies highlights the progressive risk of malnutrition in this population. For instance, in a 12-month cohort 9% of patients classified as being malnutrition risk-free at baseline using the MUST ultimately developed malnutrition according to ESPEN criteria [108]. In parallel, as many as 77% of those categorized as being of high-malnutrition risk became malnourished over the same period of time [84]. However, while MUST effectively distinguishes high-risk patients, its ability to stratify those at moderate risk is weaker and in poor agreement with the GLIM criteria [92] and the Subjective Global Assessment (SGA) tool [96]. The incorporation of anthropometric metrics in the nutritional assessment within this medium-risk category could facilitate more accurate stratification [92]. However, these assessments do not constitute part of the standard clinical practice [92]. The prognostic significance of malnutrition in SSc is striking, as increased risk for malnutrition correlates with greater mortality risk [89,90,92,99].

Patients with malnutrition also presented greater sarcopenia prevalence [87], reduced body cell mass [94,95], and decreased bone mineral density at the spine, femur [80], arms, legs, trunk, and pelvis [79]. As expected, GI involvement is persistent in malnutrition, presenting vomiting and dysphagia [92], GERD, chronic intestinal pseudo-obstruction, reduced oral aperture, decreased interincisal distance [85], abdominal distension [86], bowel involvement [103], malabsorption [86], and increased GI complaints [86,92]. The latter may include reflux symptoms, swallowing difficulties, early satiety, and retrosternal burning, all of which can interfere with adequate food intake [86].

Furthermore, malnutrition in SSc has been associated with cardiac [85] and/or lung involvement, pulmonary hypertension [106], and limited forced vital capacity (FVC) [94,106] or diffusing capacity of the lung for carbon monoxide (DLCO) [106]. Regarding SSc manifestation, patients presented higher disease severity [81,82,86], longer disease duration [80], and had diffuse skin involvement [86,87]. On the other hand, greater MUST scores were also associated with shorter disease duration [86]. Those who were at moderate-to-high risk for malnutrition tended to exhibit less frequent antinuclear antibodies (ANA) and anti-centromere positivity compared to patients at low risk for malnutrition [106]. Patients with mild and moderate malnutrition, as assessed by the CONUT, had higher C-reactive protein (CRP) levels and red-cell-distribution-width (RDW), lower hemoglobin concentrations, signs of increased inflammation, and nutrient deficiencies [93].

Beyond organ-specific morbidity and mortality, malnutrition profoundly impairs daily life. Patients report worse scores on the 36-item short form (SF-36) health survey [92,98], health assessment questionnaire disability index (HAQ-DI) [92], and disease-specific QoL measures [98]. They also present worse emotional well-being [80,96]. Even dietary intake patterns differ, with individuals at low malnutrition risk consuming relatively more carbohydrates and patients at high risk adhering to a more restricted dietary pattern, reflecting both disease-driven and behavioral adaptations [72]. Patients at greater risk for malnutrition also demonstrate poorer physical activity [92] and are more frequently treated with steroids [80] or immunosuppressants [92].

More contemporary measures, such as phase angle (PhA)—a proxy for nutritional and hydration status—were associated with greater modified Rodnan Skin Score (mRSS), poorer nutritional status, lower FVC values, and increased inflammation, as determined by the erythrocyte sedimentation rate (ESR) [94].

### Possible Factors Underlying Malnutrition and GI Involvement

Several clinical and serological factors influence malnutrition risk and GI complications in SSc. Longer disease duration has been associated with greater cumulative GI damage and greater nutritional compromise [80], while the diffuse cutaneous subtype more often presents with earlier and more severe GI involvement compared to the limited disease subtype [86,87]. Autoantibody profiles also appear relevant: Scl-70 positivity has been identified as a predictor of malnutrition [84], consistent with its associations with capillary rarefaction, interstitial lung disease (ILD), and more severe disease [106]. Patients with moderate-to-higher malnutrition risk seem to have less ANA and anti-centromere positivity compared to those at low malnutrition risk [106]. Microvascular abnormalities, particularly the “late” scleroderma pattern on nailfold capillaroscopy and capillary rarefaction, are related to both malnutrition and severe GI disease, independently of disease duration [106]. Additionally, cardiopulmonary involvement, including pulmonary hypertension and extensive ILD, frequently occurs in parallel to malnutrition, further contributing to the overall disease burden [92,106].

## 5. Sarcopenia in SSc

Reduced muscle mass and function in SSc are a consequence of genetic predisposition [109] and inflammatory processes, with the release of specific cytokines—including interleukin-6 (IL-6) and tumor-necrosis factor-α (TNF-α)—that disrupt muscle turnover and promote muscle degradation [110]. Μedications prescribed for the management of SSc, including glucocorticoids [111], propel muscle catabolism, leading to muscle deterioration [112,113]. Although many sarcopenia definitions have been suggested, no gold standard has been established, particularly in the context of rheumatic and musculoskeletal disorders, resulting in a wide reported prevalence range (Table 4) [114].

The literature suggests that, in the context of SSc, sarcopenia affects 5% [122] to 52.9% [118] of patients, while the risk of sarcopenia ranges between 20–22% [116,122]. The use of the SARC-F (strength, assistance with walking, rise from a chair, climb stairs, and falls) questionnaire, adjusted for age and body mass (SARC-F+EBM) has been suggested [116] as having greater sensitivity in scleroderma, compared to solely using the SARC-F [125].

Patients with SSc and sarcopenia have a greater chance of being malnourished and tend to have longer disease duration compared to the rest, indicating that disease progression might propel the development of sarcopenia [80]. Age, disease duration, malnutrition, fat mass, mRSS, capillaroscopy score, esophageal involvement, ESR, and extractable nuclear antigen (ENA) positivity are greater in sarcopenic patients, while DLCO is lower [13]. With respect to the clinical manifestations, SSc with sarcopenia presents diminished capillary density and impaired peripheral circulation [117], as well as more pronounced involvement of the lungs and skin [80]. Reduced skeletal mass has been related to disease duration, higher mRSS and ESR, esophageal involvement, ANA positivity, lower DLCO [13], and bone mineral content [117]. Osteoporosis is also apparent [127], increasing the risk for vertebral fractures [128], hand deformities, and disability [129]. Elevated CRP levels have also been documented, as the residue of increased inflammation status [120]. Furthermore, patients diagnosed with SSc and sarcopenia concurrently exhibit an elevated mortality rate, which escalates tenfold in cases of hospitalization [130].

Evidence from multiple studies links sarcopenia to reductions in overall physical performance [12,122], as shown by handgrip strength [12,13], gait speed [116,119,120,122], and the sit-to-stand test [115,122]. Impaired muscle mass [13,121] may result from both inflammatory and oxidative status, but also from lifestyle factors, leading to muscle wasting [12]. Sarcopenia is frequently misinterpreted as a consequence of being underweight, while recently the term sarcopenic obesity emerged [131], changing this notion. This condition is characterized by the simultaneous presence of reduced muscle mass, strength, and function, concurrent with a body mass index (BMI) that exceeds 30 kg/m^2^ [131]. According to the literature, 3.6% [106] to 24.1% of patients with SSc exhibit low BMI (<18.5 kg/m^2^), indicative of being underweight (Table 2). Nonetheless, obesity is also apparent in this population, with a prevalence of 4.8% [94] to 24.2% [102], thus, the assessment of reduced muscle mass and function should not be ruled out. Recent research indicates that sarcopenic obesity is prevalent among patients with rheumatic diseases and is associated with diminished health outcomes and an increased vulnerability to malnutrition [114].

### Frailty in SSc

Reduced muscle mass, strength, and endurance—together with weakness and weight loss—contribute to the development of frailty, a condition affecting more than half of patients with SSc [17] and SSc-associated ILD (SSc-ILD) [132]. Frailty has been associated with higher scores on the UCLA Scleroderma Clinical Trials Consortium Gastrointestinal Tract 2.0 (UCLA SCTC GIT 2.0) [16,133]—with dysphagia and abdominal distention identified as particularly severe contributors [133]—as well as with greater overall organ damage [17]. It is also linked to increased hospital admissions within the past year and a higher risk of malnutrition [16]. Moreover, frail patients demonstrate greater dependence in both basic activities of daily living (BADL) and instrumental activities of daily living (IADL) [16], alongside increased patient- and physician-reported visual analogue scale (VAS) scores and a reduced QoL [16]. Taken together, these findings suggest that frailty imposes substantial limitations on daily functioning, ultimately leading to impaired QoL and increased morbidity.

## 6. Nutrient Deficiencies

Due to SSc-related clinical manifestations, including GI involvement and malabsorption, patients often exhibit significant nutrient deficiencies [134]. Iron deficiency, first reported over 50 years ago [135], affects approximately one in four patients with SSc, even in the absence of anemia [136]. Patients with scleroderma have also been shown to attain low levels of ascorbic acid, α-tocopherol, Selenium, and carotene [7,137]. When coexisting with pulmonary hypertension, Iron deficiency further increases the risk of mortality and physical dysfunction [138]. Similarly, decreased Zinc levels are observed in patients with autoimmune diseases [139], impairing both innate and adaptive immune responses, weakening pathogen clearance, and disrupting immune regulation [18]. With regard to Selenium deficiency, research indicates that it affects 15.6% of patients [139] and is associated with an increased risk of atherosclerosis [82] and tissue fibrosis [140].

**Figure 1:**
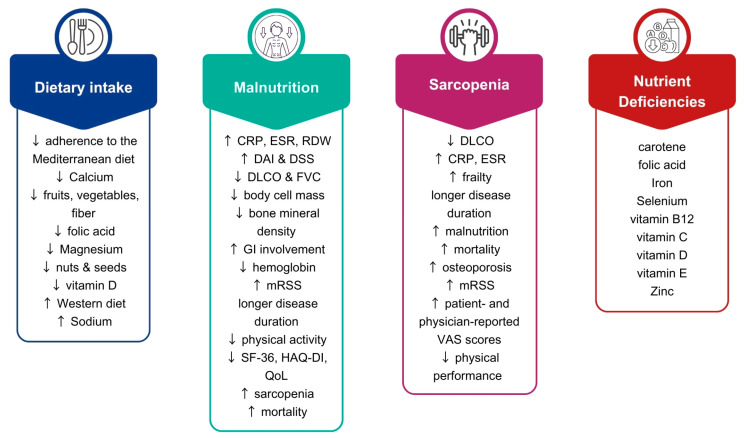
Implications for nutritional status and clinical outcomes in SSc. CRP: C-reactive protein; DAI: disease activity index; DSS: disease severity score; DLCO: diffusing capacity of the lung for carbon monoxide; ESR: erythrocyte sedimentation rate; FVC: forced vital capacity; GI; gastrointestinal; HAQ-DI: Heath assessment questionnaire disability index; mRSS: Modified Rodnan Skin Score; QoL: Quality of life; RDW: Red-cell-distribution-width; SF-36: 36-item short form health survey; VAS: Visual analogue scale; ↑ increased; ↓ decreased.

Patients with SSc who develop malnutrition frequently exhibit deficiencies in essential micronutrients, including folic acid [55], vitamin B12 [55,102], Iron [55], vitamin D [141], and vitamin C [82]. Serum vitamin D levels have been inversely correlated with SSc severity, while vitamin C deficiency has been linked to higher mRSS, as well as esophagitis, or Barrett’s mucosa [141]. Furthermore, patients presenting with at least one micronutrient deficiency were more likely to exhibit skin fibrosis proximal to the metacarpophalangeal joints, secondary joint contractures, higher median mRSS, and elevated ESR levels [139]. Figure 1 provides an overview of the effects of SSc on nutritional status and nutrition-related clinical outcomes in patients with SSc.

## 7. Specific Nutrients and Dietary Patterns

The European League Against Rheumatism (EULAR) recommends adopting a healthy lifestyle, including proper nutrition and regular physical activity, to help prevent the progression of SSc [142]. Alongside these lifestyle measures, certain nutrients with anti-inflammatory and antioxidant properties have shown potential benefits, although research in this area remains limited. Omega-3 fatty acids [143,144], vitamin D [145], Selenium [146], Magnesium [147], monounsaturated fatty acids [148], and probiotics [149] all demonstrate anti-inflammatory effects, underscoring the potential value of immunonutrition through personalized dietary interventions [150]. Therapeutic dietary patterns such as the MedDiet [151,152] and the Autoimmune Protocol diet (AIP) [153] may confer beneficial effects, although the evidence remains limited.

Emerging studies on dietary interventions have shown contradictory results in SSc. Patients with SSc who fail to adhere to the MedDiet tend to experience a greater disease burden, including digestive involvement, impaired physical function and activity, sarcopenia [154], reduced QoL, depression, anxiety, as well as GI and vascular symptoms [129,155]. The use of probiotics in SSc has yielded mixed results. Some studies reported improvements in the UCLA SCTC GIT 2.0 score and related symptoms, such as reflux, bloating, and emotional distress [156], while others found no effect after 8 weeks of supplementation [157,158]. However, prolonged use, with a 120-day high-dose multi-strain probiotic intervention, led to significant improvement in GIT-reflux scores—though not in total GIT scores—and was associated with trends toward increased microbiota diversity [157]. Fructose [159] and lactose malabsorption [160] have also been reported in SSc. However, adherence to a low FODMAP diet has not been associated with improvements in GI symptoms [161]. Regardless of dietary patterns, patients with SSc who have impaired GI function often develop malabsorption, which is followed by severe malnutrition and low muscle mass [7]. Medical nutrition therapy, encompassing higher calorie and protein intake, texture-modified foods, and lifestyle adjustments, alleviated symptom burden and may have promoted appendicular lean mass [91]. A 12-week high-energy, high-protein oral supplementation improved SGA and hand grip strength, underscoring the value of routine nutritional assessment and targeted intervention in this population [162]. Nevertheless, both supplemental treatment and parenteral nutrition improved the nutritional status of patients with malnutrition, with improvements in PhA [94].

Both enteral and parenteral nutrition are employed in SSc, depending on disease severity [163,164]. Oral nutritional supplements (ONS), with or without dietary counseling, have also been used in patients at risk of malnutrition [165]. Nevertheless, tolerance to ONS and enteral feeding can be limited in individuals with advanced fibrosis or malabsorption, who may eventually progress to intestinal failure [166]. In such cases, parenteral nutrition may become necessary, although this approach carries recognized risks. Reported adverse events include refeeding syndrome in profoundly malnourished patients at the start of therapy, metabolic imbalances such as elevated triglycerides and blood glucose, bone complications including osteomalacia and osteoporosis, and hepatobiliary problems such as fatty liver, cholestasis, cholecystitis, and gallstones [167].

A systematic review indicated that ONS and dietary counseling in SSc appear to have limited efficacy, as they do not significantly improve weight, BMI, energy, and protein intake, or QoL [166]. Nonetheless, ONS may contribute to reducing malnutrition risk and alleviating nutrition-impact symptoms, even though they fail to influence GI manifestations or disease progression [166]. The available evidence for enteral nutrition is limited; however, case reports have suggested the potential for increases in weight and BMI [166]. Parenteral nutrition, particularly when delivered at home, has shown more consistent effects, with improvements in BMI, weight, and QoL, but without measurable impact on disease progression, survival, or functional capacity [166].

## 8. Nutrition Recommendations by Scientific Societies

According to the North American Expert Panel convened by the Canadian Scleroderma Research Group [77], malnutrition is a pivotal comorbidity in SSc. For this, all patients should be frequently screened in order to improve disease prognosis and QoL. In Australia, rheumatologists commonly agree that dietetics services would be advantageous in supporting their patients to gain weight and manage SSc symptomatology [73]. The Michigan Medicine Scleroderma Program [38] advocates for dietary and lifestyle changes that may benefit patients with SSc, including general dietary advice, as well as recommendations for managing specific complications or symptoms. On the other hand, the UK Scleroderma Study Group [71] has issued recommendations for managing GI disorders in scleroderma. Similarly, more recently, Scleroderma Australia has published a booklet, aiming to assist patients with nutritional concerns relating to scleroderma [39]. Apart from these, and despite the importance of nutrition in scleroderma, there is a lack of recommendations developed by scientific societies on addressing the nutritional requirements of patients with SSc. Table 5 summarizes the available recommendations produced by the aforementioned scientific societies/institutes.

## 9. Conclusions

GI involvement is one of the most significant manifestations of SSc, affecting more than two-thirds of patients. These complications often interfere with daily life and can influence disease outcomes. Inadequate nutrition and reduced food intake may result in malnutrition, sarcopenia, and frailty, while malabsorption further worsens patients’ overall health. The consequences are substantial, contributing to increased mortality and higher healthcare costs. Addressing these challenges requires close interdisciplinary collaboration, and routine nutritional assessment should be considered essential in hospital care. Moreover, there is a clear need for consensus guidelines specifically adapted to the unique requirements of individuals with SSc. This is crucial for delivering optimal, personalized, and patient-centered management.

## 10. Future Directions and Limitations

Although many studies have highlighted the association between GI impairment and adverse clinical outcomes, nutritional assessment has yet to find its place in routine screening. Patients with SSc frequently present malnutrition, sarcopenia, and loss of muscle mass, all of which contribute to reduced QoL and impair psychological as well as social well-being [168]. Difficulties related to eating—including reflux, dysphagia, early satiety, and impaired motility—further exacerbate inadequate nutrient intake and increase the risk of multiple micronutrient deficiencies. These deficiencies are not merely secondary complications; they have been shown to significantly affect disease progression, functional decline, and, ultimately, mortality [19].

Functional limitations also contribute significantly to the augmented nutritional risk in SSc. Hand deformities, digital ulcers, and severe Raynaud’s phenomenon can impair manual dexterity and grip strength, making grocery shopping, food handling, and meal preparation particularly challenging for some patients [73]. These barriers may reduce both the variety and quality of the diet, promote dependence on others for food-related activities, and contribute to unintentional weight loss or inadequate nutrient intake. Importantly, such limitations are not captured by most malnutrition screening tools, yet they represent a pragmatic challenge to maintaining adequate nutrition and independence in daily living.

Beyond their clinical and functional impact, nutritional impairments in SSc lead to increased rates of hospitalization, recurrent infections, and functional disability, contributing substantially to the healthcare and socioeconomic burden [169]. Healthcare providers must therefore be better informed about the nutritional challenges apparent in SSc and trained in the identification, screening, and management of these conditions. As this isn’t always possible, interdisciplinary collaboration becomes essential to deliver high-quality, patient-centered care. Dietitians trained in SSc will play a pivotal role in evaluating and optimizing the nutritional status of these patients, supporting both clinical management and dietary modifications tailored to their specific needs. Their integration within rheumatology and specialized SSc clinics, ideally through dedicated immunonutrition units, has the potential to strengthen multidisciplinary care. Future research should explore how such dietary approaches can be optimally integrated with other interventions, such as pharmacological treatments, physical activity [170], and stress management, to maximize therapeutic outcomes and long-term health.

## Figures and Tables

**Table 1:** Clinical manifestation of SSc in the GI tract.

GI Tract Involvement	Clinical Manifestations
Oral cavity	Microstomia, xerostomia, dental caries, periodontal disease, dystrophic calcinosis, bone/joint involvement; worsened by coexisting Sjögren’s syndrome; reflux increases acidity, dental wear.
Esophagus	Dysmotility disorder, decreased peristalsis, hypotensive LES, dysphagia, regurgitation, heartburn, GERD—up to 76% prevalence.
Stomach	Gastritis, gastroparesis, gastric ulcers, GAVE. Symptoms: abdominal pain, bloating, early satiety, nausea. GAVE linked to anemia/bleeding.
Small intestine	Dysmotility (40–88%), pseudo-obstruction, SIBO (weight loss, malabsorption, dysbiosis), rare PCI, possible celiac disease.
Colon	Hypomotility (20–50%), colonic dilatation, diarrhea (SIBO, malabsorption), constipation, fecal incontinence (anorectal dysfunction, internal anal sphincter involvement).

GAVE: gastric antral vascular ectasia; GERD: gastroesophageal reflux disease; GI: gastronintestinal; LES: lower esophageal sphincter; PCI: pneumatosis cystoides intestinalis; SIBO: small intestinal bacterial overgrowth; SSc: systemic sclerosis.

**Table 2:** Studies assessing malnutrition in patients with SSc.

First Author	Recruitment		Sample		BC Method Applied	Malnutrition Criteria Applied	Obesity (%)	Underweight (%)	Nutritional Risk (Other Tools) (%)			Low FFMI (%)
	Site	Duration	Size (N)	Men/Women Ratio					Low	Moderate	High	
Andréasson [55]	Skane University Hospital, Lund	2014–5	98	NR	-	MUST	-	-	80.6^MUST^	19.4^MUST^		-
Bagnato [84]	University Hospital of Messina and Padova and University of Leeds	NR	159	11/148	-	MUST, ESPEN	-	-	Discovery cohort 45^MUST^, validation cohort 47^MUST^, 84.3^ESPEN^	Discovery cohort 37^MUST^, validation cohort 34^MUST^	Discovery cohort 18^MUST^, validation cohort 18^MUST^	-
										15.7^ESPEN^		
Baron [86]	15 centers, Canada	2004–8	586	76/510	-	MUST	-	-	70.1^MUST^	12.5^MUST^	17.4^MUST^	-
Burlui [72]	Dept of Rheumatology and Rehabilitation, “Grigore T. Popa” University of Medicine	NR	42	6/36	-	MUST	-	-	73.81^MUST^	11.9^MUST^	14.3^MUST^	-
Caimmi [80]	Verona Medical School	2016	141	22/119	DXA	ESPEN, MUST	-	-	79.4^MUST^, 90.8^ESPEN^	12.8^MUST^	7.8^MUST^	-
										9.2^ESPEN^		
Cano-Garcia [87]	Regional Universitario de Málaga	NR	52	1/51	-	ΜΝA	-	-	64.5^MNA^	32.7^MNA^	1.9^MNA^	-
Caporali [88]	Rheumatology Unit, Research Hospital Fondazione IRCCS Policlinico San Matteo	2007–8	160	20/140	-	BMI, weight loss	-	-	85	15	-	-
Cereda [89]	Rheumatology Unit, Fondazione IRCCS Policlinico San Matteo	2007–8	160	20/140	-	MUST	-	3.1	45.6^MUST^	30^MUST^	24.4^MUST^	-
Cruz-Domínguez [90]	Hospital (NOD) in Mexico City	2005–14	147	NR	-	Chang index	-	-	Survivors 64^Chang^, Deceased 32^Chang^	Mild: Survivors 4.7^Chang^, Deceased 5.3^Chang^; Moderate: Survivors 18.8^Chang^, Deceased 10.5^Chang^	Survivors 12.5^Chang^, Deceased 52.6^Chang^	-
Doerfler [91]	Multiple sites, N. America, Europe	NR	14	12/2	DXA	abPGSGA	-	-	83^abPGSGA^ *	17^abPGSGA^ *		-
Dupont [82]	Toulouse University Hospital	2011–6	82	20/62	-	HAS, MUST	-	-	83^HAS^, 79^MUST^	6^MUST^	15^MUST^	-
										17^HAS^		
Fairley [92]	Multicenter trial, Australia	2007–23	1903	272/1631	-	GLIM, MUST	-	-	43.3^MUST^, 20.9^GLIM^	25.9^MUST^, 46^GLIM^	30.7^MUST^, 33.1^GLIM^	-
Gajdecki [93]	Rheumatology Dept, Medical University of Łódź	2018–23	44	34/10	-	CONUT, MUST	4.9	9.8	65.9^MUST^, 38.6^CONUT^	19.5^MUST^, light: 43.2^CONUT^, moderate: 43.2^CONUT^	14.6^MUST^, 20.9^GLIM^, 0^CONUT^	-
Hax [16]	Hospital de Clínicas, Porto Alegre	2019	94	7/87	-	ESPEN	-	-	85.1^ESPEN^	14.9^ESPEN^		-
Hvas [78]	Rheumatology specialist center, Salford	NR	168	31/137	BIA	BMI, MUST	11	7	74^MUST^	14^MUST^	12^MUST^	-
Krause [94]	Dept of Rheumatology & Clinical Immunology, Charitéplatz	NR	124	20/104	BIA	BMI, PhA	4.8	13.7	44.4^PhA^	33.8^PhA^	21.8^PhA^	-
Molfino [95]	Scleroderma Unit, Dept of Translational & Precision Medicine, Azienda Policlinico Umberto I, Sapienza University	NR	64	11/55	BIA	MUST, FFMI, BMI	NR	NR	61^MUST^	12.5^MUST^	26.5^MUST^	28.2
Murtaugh [96]	University of Utah SSc Center	NR	24	4/20	-	MUST, SGA	-	-	50^SGA^, 62.5^MUST^	37.5^SGA^, 8.3^MUST^	12.5^SGA^, 29.2^MUST^	-
Ortiz-Santamaria [97]	Hospital Universitario de Granollers	NR	9	1/8	-	MUST	NR	0	87.5^MUST^	12.5^MUST^		-
Paolino [79]	Scleroderma Clinic, Rheumatology Division, Genova University	NR	36	0/36	DXA	ESPEN	NR	-	63.8^ESPEN^	36.1^ESPEN^		NR
Preis [98]	Dept of Rheumatology & Clinical Immunology, Charité—University Medicine Berlin	2013–4	129	12/117	-	BMI, MUST	NR	6.2	74.4^MUST^	14.7^MUST^	10.9^MUST^	-
Rivet [85]	Dept of Internal Medicine, Saint Eloi University Hospital	1985–2019	119	18/101	-	HAS, MUST	-	-	40.8^HAS^, 41.7^MUST^	34.2^HAS^, 20^MUST^	25^HAS^, 38.3^MUST^	-
Rosato [81]	Dept of Translational & Precision Medicine, Sapienza University	2018	102	16/86	BIA	MUST, ESPEN, GLIM, FFMI	-	-	69.6^MUST^, 83.4^GLIM^ 91.2^ESPEN^	12.7^MUST^, 12.7^GLIM^	17.6^MUST^, 3.9^GLIM^	21.6
										8.8^ESPEN^		
Rosato [99]	Dept of Translational & Precision Medicine, Sapienza University	2017–21	101	15/86	-	BMI, GLIM	-	-	78.2^GLIM^	12.9^GLIM^	8.9^GLIM^	-
Rosato [100]	Dept of Translational & Precision Medicine, Sapienza University	ΝR	104	16/88	BIA	MUST, GLIM	-	-	69.2^MUST^, 79.8^GLIM^	10.6^MUST^, 11.5^GLIM^	20.2^MUST^, 8.7^GLIM^	-
Spanjer [101]	Dept of Rheumatology, Amsterdam Rheumatology & Immunology Center	2013–4	72	21/51	BIA, DXA	FFMI, BMI	11.1	4.2	91.7^ESPEN^	8.3^ESPEN^		20.8
Szabo [83]	County Emergency Clinical Hospital Cluj-Napoca	NR	75	NR	-	MUST	-	-	93^MUST^	6^MUST^	1^MUST^	-
Tas Kilic [102]	Ankara Numune Training & Research Hospital, Ankara	NR	62	6/56	-	BMI, MUST	24.2	11.3	74.2^MUST^	25.8^MUST^		-
Türk [103]	Division of Rheumatology, Cukurova University, Adana	2016–7	98	15/83	-	MUST	-	-	61.2^MUST^	15.3^MUST^	23.5^MUST^	-
Pardali (in press)	Dept of Rheumatology & Clinical Immunology, Larissa University Hospital	2022–5	29	4/25	Skinfolds	GLIM, SGA	20.7	24.1	82.8^SGA^, 27.6^GLIM^	6.9^SGA^, 37.9^GLIM^	10.4^SGA^, 34.5^GLIM^	NR
Volkmann [1]	UK hospital (NOD)	2015–7	576	143/433	-	MUST	-	10.6	Νintedanib 91.7^MUST^; control 87.2^MUST^ *	Νintedanib 5.9^MUST^; control 8^MUST^ *	Νintedanib 2.4^MUST^; control 4.9^MUST^ *	-
Wojteczek [104,105]	Clinical University Centre, Gdańsk	2013–4	56	9/47	BIA	ESPEN, GLIM, 7-SGA, SNAQ	12.5	5.4	82.1^ESPEN^, 76.8^7-SGA^, 83.9^SNAQ^, 37.5^GLIM^	21.4 ^7-SGA^, 50^GLIM^	1.8^7-SGA^, 12.5^GLIM^	73.2
										16.1^SNAQ^, 17.9^ESPEN^		
Yalcinkaya [106]	Division of Rheumatology, Marmara University	NR	134	10/124	-	BMI, MUST	NR	3.6	85^MUST^	9^MUST^	6^MUST^	-

abPGSGA: abridged Patient-Generated Subjective Global Assessment; BC: Body composition; BIA: Bioelectrical impedance analysis; BMI: Body mass index; DXA: dual-energy X-ray absorptiometry; Chang index [107]; CONUT: Controlling Nutritional Status; ESPEN: European Society for Clinical Nutrition and Metabolism; FFMI: fat-free mass index; GLIM: Global Leadership Initiative on Malnutrition; HAS: National Health Authority; MNA: Mini Nutritional Assessment; MUST: Malnutrition Universal Screening Tool; NOD: not-other defined; NR: not reported; PhA: Phase angle; SGA: Subjective Global Assessment; SNAQ: Short Nutritional Assessment; SSc: Systemic Sclerosis. * baseline data.

**Table 3:** Malnutrition assessment methods and their limitations when applied to patients with SSc.

Malnutrition Assessment Method	Components	Limitations of Methods When Applied in SSc
abPG-SGA	Patient-reported intake, symptoms, weight, activity.	– Symptom overlap with disease activity (e.g., reflux, early satiety, bloating) complicates scoring. – Patient recall bias may affect reliability. – Physical activity reduction may reflect musculoskeletal limitations, not malnutrition per se.
BMI	Weight/height.	– Poor reflection of muscle and fat distribution. – Can be normal despite severe sarcopenia or malabsorption. – Edema, skin thickening, or calcinosis may distort accuracy.
Chang index	Nutritional risk using parameters like weight loss, BMI, GI symptoms, functional status.	– GI symptoms in SSc are highly variable (esophagus, stomach, small bowel, colon involvement). – Index was not validated in SSc; may underestimate severity in patients with severe dysphagia or pseudo-obstruction.
CONUT	Albumin, total cholesterol, lymphocyte count.	– Albumin and cholesterol may be affected by inflammation, not nutrition alone. – Lymphopenia may result from immunosuppressive therapy rather than malnutrition. – Risk of overestimating malnutrition due to non-nutritional factors.
ESPEN guidelines	– BMI-based cutoffs may underestimate risk. – Muscle mass assessment not always feasible in routine rheumatology clinics. – Overlooks disease-specific GI factors (e.g., dysmotility, malabsorption).	– BMI-based cutoffs may underestimate risk. – Muscle mass assessment not always feasible in routine rheumatology clinics. – Overlooks disease-specific GI issues (e.g., dysmotility, malabsorption).
FMI	Fat mass adjusted for height.	– Fat mass alone does not reflect muscle status (sarcopenic obesity possible). – BIA/DXA accuracy can be limited by skin and soft tissue changes.
GLIM	Phenotypic (weight loss, low BMI, reduced muscle mass) + etiologic (inflammation, reduced intake/assimilation).	– Muscle mass assessment again is challenging. – Inflammation in SSc is variable and may not correlate with nutritional risk. – Does not capture oral and esophageal dysfunction, key drivers of malnutrition in SSc.
HAS	Weight loss, BMI, acute disease effect.	– Similar to MUST, BMI- and weight-based tools can miss sarcopenia. – Does not consider chronic GI dysfunction common in SSc.
MNA	– Anthropometry: BMI, weight loss, mid-arm circumference, calf circumference. – Dietary assessment: Food intake decline, number of meals, fluid intake, protein sources. – General assessment: Lifestyle, medication, mobility, psychological stress. – Subjective assessment: Self-perceived health and nutrition.	– Anthropometric bias: Skin thickening, edema, and calcinosis distort BMI and circumference measures. – Mobility assessment confounding: Reduced mobility in SSc may be musculoskeletal or vascular, not nutritional. – Appetite vs. swallowing: Appetite may be intact, but chewing/swallowing dysfunction reduces intake—MNA doesn’t differentiate. – Focus on elderly populations: Originally validated in geriatric cohorts, not in SSc or autoimmune diseases, so cutoffs may not apply. – Time-consuming: Longer to administer than shorter screens.
MUST	BMI, unintentional weight loss, acute disease effect.	– Weight loss may be masked by edema, ascites, or skin thickening. – BMI may not reflect actual body composition (e.g., sarcopenia in normal-BMI patients). – Acute disease effect criterion is less applicable since malnutrition in SSc is often chronic.
PhA	Cell membrane integrity, body composition.	– Bioimpedance accuracy may be compromised by skin thickening, edema, or fibrosis. – Not widely available in routine SSc care.
SGA	Clinical judgment integrating history and physical exam.	– Subjective; dependent on assessor experience. – GI symptoms are disease-related, making interpretation tricky. – Muscle wasting may be underestimated due to skin/fibrosis masking.
SNAQ	– Unintentional weight loss (≥4 kg in 6 months/≥2 kg in 1 month). – Appetite reduction. – Need for supplemental drinks or tube feeding.	– Weight loss may be masked by edema or skin thickening, leading to underestimation. – Appetite is not always reduced in SSc—many patients have adequate appetite but can’t eat normally due to dysphagia, reflux, or early satiety → underestimates malnutrition. – Supplemental feeding use is not universal even in severe cases, so patients may score “low risk” despite clinically relevant malnutrition. – Focuses mainly on intake/weight but ignores malabsorption, GI dysmotility, or sarcopenia, all common in SSc.

abPGSGA: abridged Patient-Generated Subjective Global Assessment; BIA: Bioelectrical impedance analysis; BMI: Body mass index; DXA: dual-energy X-ray absorptiometry; Chang index; CONUT: Controlling Nutritional Status; ESPEN: European Society for Clinical Nutrition and Metabolism; GI: gastrointestinal; GLIM: Global Leadership Initiative on Malnutrition; HAS: National Health Authority; MNA: Mini Nutritional Assessment; MUST: Malnutrition Universal Screening Tool; PhA: Phase angle; SGA: Subjective Global Assessment; SNAQ: Short Nutritional Assessment; SSc: Systemic Sclerosis.

**Table 4:** Studies assessing sarcopenia in patients with SSc.

First Author	Sample		Sarcopenia		
	N	Prevalence (%)	Diagnostic Criteria	Sarcopenia Assessment Methods	Muscle Mass Assessment Methods
Ajdynan [115]	43	33.3	EWGSOP 2019	SMI ^‡^, HGS, sit-to-stand	DXA
Caimmi [80]	140	20.7	SMI	SMI ^‡^	DXA
Corallo [13]	62	42	EWGSOP 2010	SMI ^‡^, HGS	DXA
Doerfler [91]	13	54	SMI	SMI ^‡^	DXA
Hax [116]	94	15.9/22.3/ 21.3/21.3/36.2	EWGSOP 2019/SARC-F/ SARC-CalF/SARC-F+EBM/Ishii	SMI ^‡^, HGS, 4MWS, SPPB	DXA
Paolino [117]	43	23.3	EWGSOP 2010	SMI ^‡^	DXA
Pardali [118]	17	52.9	EWGSOP 2010	FFMI ^¤^, HGS	Skinfold thickness
Rincón [119]	27	33.3	EWGSOP 2010	SMI ^‡^, HGS, 4MWS	DXA
Sangaroon [120]	180	22.8	AWGS 2019	SMI ^‡^, FFMI ^¤^, HGS, 6MWS	DXA
Sari [121]	93	10.7	EWGSOP 2010	ASMI ^†^, HGS	BIA
Siegert [12]	129	22.5	EWGSOP 2010	SMI ^‡^, HGS	BIA
Yuce Inel [122]	80	20/5/20/8.8	SARC-F/SMI ^‡^/SMMI ^Š^/FFMI ^¤^	4MWS, sit-to-stand	BIA

ALM: appendicular lean mass; ASMI: appendicular skeletal muscle mass index; AWGS: Asian Working Group for Sarcopenia [123]; BIA: bioelectrical impedance analysis; BW: body weight; DXA: dual-energy X-ray absorptiometry; EWGSOP: European Working Group on Sarcopenia in Older People [124,125]; FFM: fat-free-mass; FFMI: fat-free mass index; HGS: handgrip strength; Ishii: estimate the probability of sarcopenia included three variables: age, grip strength, and calf circumference [126]; kg: kilogram; m: meter; MWS: meter walking speed; NR: not reported; SARC-F: strength, assistance with walking, rise from a chair, climb stairs, and falls; SARC-CalFL: SARC-Calf combining calf circumference; SARC-F+EBM: SARC-F adding age and body mass; SMI: skeletal muscle index; SPPB: short physical performance battery; SSc: systemic sclerosis; ^†^ ASMΙ: ALM/height^2^ where ALM = −4.211 + (0.267 × height^2^/resistance) + (0.095 × BW) + (1.909 × sex [men = 1, women = 0]) + (−0.012 × age) + (0.058 × reactance); ^¤^ FFMI: FFM divided by the square of the height (kg/m^2^); ^‡^ SMI: the sum of upper and lower limb muscle mass (ALM) divided by squared height (kg/m^2^); ^Š^ SMMI = SMM/height^2^, where SMM = FFM × 0.566.

**Table 5:** SSc-specific nutritional recommendations by scientific societies/institutes.

Key Points	Details/Criteria
Dietary practices	Educate patients on safe dietary modifications. Prevent overly restrictive diets that worsen malnutrition or deficiencies. Support individualized dietary advice rather than patient-led restrictions [73].
Dietetic referral & service delivery model	Refer to a dietitian ideally at diagnosis, at symptom change, and periodically thereafter. A dietitian should have SSc expertise/experience; multidisciplinary care is optimal [73,77]. Provide dietetic input through written resources, face-to-face, telehealth, phone calls, online resources, and group sessions [73].
Symptom screening & monitoring	Screen all patients with SSc at baseline and monitor regularly, as this strongly affects nutrition and QoL [73]. Early intervention reduces complications [73]. BMI < 18.5 kg/m^2^ suggests PEM [73].
Weight loss & malnutrition	Substantial weight loss over 3–6 months may indicate inadequate intake [38]. Implement systematic malnutrition screening (MUST or similar). Monitor weight regularly [38,73]. Rule out bacterial overgrowth and gastroparesis [38]. Screen for malabsorption, PBC, pancreatic insufficiency [71]. Increase healthy fats (olive/canola/peanut oil, nuts, seeds, avocado, fatty fish, coconut milk, oil-based dressings) [39,71]. Use calorie-dense smoothies (fruit + yogurt + milk + nut butter + oil + protein powder). Increase protein (marinate meats to soften; use broths; add protein powders to smoothies, cereal, yogurt) [39] and add high-protein supplements [71]/Eat every 2 h for maximal intake [38]. Enteral feeding if possible (jejunal/gastrostomy). Parenteral nutrition if intestinal failure. Multidisciplinary approach (rheumatology, gastroenterology, dietetics) [71].
Labs for screening to detect deficiencies and malnutrition	Hemoglobin (Iron, folate, B12), serum carotene (fat malabsorption), serum folate (↑ in bacterial overgrowth, not valid with supplements), serum albumin (<35 g/L → rule out PEM, but not sensitive/specific) [77]. Protein status: total protein, albumin, pre-albumin. Vitamin/mineral deficiencies: Iron, ferritin, TIBC, Zinc, B12. Malabsorption/SIBO: folate, carotene, vitamin D [38], serum methylmalonic acid, Zinc, vitamin D, vitamin K or prothrombin time, breath tests (C14 xylose = better, hydrogen breath = more available and often abnormal in SSc with bacterial overgrowth) [77].
Other screening	Ask about GI symptoms, oral health (teeth, chewing, taste), saliva production, depressive symptoms. Assess severity of malnutrition [77].
General diet recommendations	Eat small, frequent meals every 3–4 h (or every 2 h if underweight). Choose fresh, minimally processed foods with short ingredient lists [38]. Avoid artificial ingredients, preservatives, and hydrogenated oils. Use adapted utensils/kitchen tools; pre-cut or frozen fruit/vegetables; stock frozen meals; occupational therapy support [39].
Inflammation	Add antioxidant-rich herbs/spices: basil, rosemary, oregano, cinnamon, ginger, paprika, cayenne, turmeric, curry powder [38], and colored fruits/vegetables (dark green, orange, yellow, red, purple, blue) [38]. Limit added sugars; natural sugars in fruit/dairy are usually fine unless GI distress occurs. Watch for hidden sugars (sucrose, cane juice, fructose, syrups, honey, molasses) [38]. Include omega-3s (fatty fish, ground flaxseed, walnuts). Eat vitamin E-rich foods (nuts, seeds, olive oil). Consider vitamin D3 [38]. Consider OTC multivitamin/mineral with Ζinc, Ιron, vitamins A, D, E, K, folate, and B12. Extra supplementation may be required if deficiencies are present. Probiotics may improve bloating/distention [38].
Bone health	Calcium sources: Dairy, bone broths, leafy greens, nuts, tahini, tinned salmon/sardines. Magnesium sources: Nuts, seeds, greens, dark chocolate. Vitamin D: Sunlight + supplements (dose per individual needs). Seek professional advice [39].
Water	Drink filtered water only (avoid plastic exposure). Use glass/stainless steel. Daily target = half body weight in ounces (e.g., 150 lb → 75 oz) [38].
Daily intake	Fruit: Two–three/day; choose colorful fresh/frozen fruits; avoid gas-causing (FODMAP). Vegetables: Five–seven/day; colorful, fresh/frozen preferred; avoid high-FODMAP veggies. Proteins: Lean meats, fish (8–12 oz fatty fish weekly), eggs, nuts, beans if tolerated; avoid processed/fried meats. Milk/dairy: Two–three/day; low-fat, Greek or lactose-free if sensitive; avoid regular lactose if symptomatic. Whole grains: Three–six/day; 100% whole grains ≥3 g fiber; avoid refined flour/wheat if FODMAP-sensitive. Fats & oils: One–two/day; olive, peanut, canola, nuts, avocado; avoid trans fats, limit butter/shortening [38].
Swallowing	Refer to a speech pathologist when oropharyngeal swallowing problems are encountered [77]. Eat slowly and chew well. Use soft/pureed foods (mashed potatoes, soups, casseroles). Dip dry foods (bread, biscuits) in liquids. Drink fluids between bites. Blend/mince foods, add sauces/oils to moisten. Seek speech pathologist input [39]. Use smoothies, blended fruits/vegetables, yogurt, cottage cheese, scrambled eggs, soft meats with sauces, pasta dishes. Add whey protein or meal replacement powders to smoothies [38].
Xerostomia	Rule out Sjögren’s (serology ± biopsy). Treat with biotin, artificial saliva, or trial of pilocarpine 5 mg or Evoxac before meals [71].
Gastroparesis	If early satiety, nausea, vomiting → do radionuclide gastric emptying study, refer to gastroenterologist. Pro-motility agents might help [77].
Reflux/heartburn	Eat small, frequent meals (avoid overfilling). Do not eat 2–3 h before bedtime. Avoid trigger foods (citrus, tomato, fried/greasy foods, coffee, garlic, onions, peppermint, gas-producing foods like beans, broccoli, raw peppers, raw onions, spicy foods, carbonated drinks, alcohol). If overweight in midsection → weight loss may help. Use a wedge pillow or elevate head of bed to reduce regurgitation [38,39]. Pharmacologic: PPIs first-line; increase dose or add H2 blocker if refractory. Consider prokinetics if delayed emptying. Surgery (fundoplication) not generally recommended due to motility disorder [71,77].
Malabsorption (bacterial overgrowth)	Trial of selective antibiotic (10–21 days). If relapses: repeated monthly courses or continuous therapy. Consider probiotics after antibiotics. For refractory small bowel involvement: octreotide 50 mcg SC at bedtime (but costly, parenteral, and may impair gastric emptying/pancreatic secretions) [39,71].
Diarrhea	Treat the identified cause. Antibiotics for SIBO, pancreatic enzyme replacement if insufficient. Bile acid sequestrants if bile acid malabsorption. Consider probiotics. Symptomatic antidiarrheals if no reversible cause found [71]. Opt for soluble fiber (banana, apple, oats, prunes) [39].
Decreased GI motility/constipation	Engage in regular exercise (e.g., walking). Eat a high-fiber diet (whole grains, fruits, vegetables). Hydrate [38,39,71]. Increase daily fluid intake [38,39]. Take a probiotic or yogurt with active cultures [38]. Room-temperature drinks if Raynaud’s [39].
Incontinence (fecal soilage)	Conservative: Bowel retraining, diet modification. Medical: Antidiarrheals if loose stools. Advanced: Sacral nerve stimulation, sphincter repair (variable long-term success) [71].
Severe refractory cases	If all else fails → enteral nutrition (preferred) via jejunostomy; parenteral nutrition if necessary (but risk of sepsis, thrombosis, liver failure). Decision requires coordination (rheumatologist, gastroenterologist, dietitian) [71].
Fatigue	Eat small, frequent meals for steady energy. Stay hydrated. Engage in 30–60 min daily moderate exercise (walking, biking, pool exercise, pilates, yoga, tai chi). Get 7–8 h of sleep nightly. If Iron is low, discuss supplementation with a doctor. Take Iron pills with vitamin C-containing juice to improve absorption [38].
Poor circulation/Raynaud’s	Exercise regularly to improve blood flow. For finger ulcers → eat animal proteins rich in Zinc and Iron (beef, pork) to promote wound healing [38].
Tight, thickened skin	Eat vitamin E-rich foods (nuts, seeds, wheat germ, olive/canola/peanut oils). Consider a biotin 5 mg supplement for skin and nail health [38].
FODMAP [38]	Oligosaccharides (FOS/GOS): Avoid wheat/rye grains, legumes, onion, garlic, asparagus, cabbage, broccoli, artichokes, leeks, shallots. Choose gluten-free grains, corn, rice, oatmeal, celery, spinach, white potatoes. Disaccharides (Lactose): Avoid milk, cheese, yogurt, ice cream, custard. Choose lactose-free dairy, unsweetened almond or rice milk. Monosaccharides (Excess fructose): Avoid apples, pears, watermelon, mango, honey, agave, high fructose corn syrup, dried fruits. Choose berries, citrus, ripe banana, kiwi, pineapple, rhubarb. Polyols (Sugar alcohols): Avoid apricot, plum, peach, prunes, avocado, mushrooms, and “diet/sugar-free” products with sorbitol, mannitol, xylitol, maltitol. No safe alternatives listed—best avoided.

BMI: body mass index; FODMAP: Fermentable oligosaccharides disaccharides monosaccharides and polyols; FOS: fructo-oligosaccharides; g: gram; GERD: Gastroesophageal reflux disease; GI: gastrointestinal; GOS: galacto-oligosaccharides; kg: kilogram; L: Liter; lb: libra; m: meter; MUST: malnutrition universal screening tool; OTC: over the counter; oz: ounce; PBC: primary biliary cirrhosis; PEM: protein-energy malnutrition; PPIs: proton pump inhibitors; QoL: quality of life; SC: subcutaneous; SGA: subjective global assessment; SIBO: small intestinal bacterial overgrowth; SSc: systemic sclerosis; TIBC: total binding iron capacity.

## Data Availability

As this is not an original research item, no primary data exist. All mentioned data are already presented in the manuscript text.

## Abbreviations

The following abbreviations are used in this manuscript:

AIP	Autoimmune Protocol
ANA	Antinuclear antibodies
BADL	Basic activities of daily living
BMI	Body mass index
CONUT	Controlling Nutritional Status
CRP	C-reactive protein
DAI	Disease activity index
DLCO	Diffusing capacity of the lung for carbon monoxide
DSS	Disease severity score
dSSc	Diffuse Systemic Sclerosis
ENA	Extractable nuclear antigen
ESPEN	European Society of Clinical Nutrition and Metabolism
ESR	Erythrocyte sedimentation rate
EULAR	European League Against Rheumatism
FODMAP	Fermentable oligosaccharides, disaccharides, monosaccharides, and polyols
FVC	Forced vital capacity
GAVE	Gastric antral vascular ectasia
GERD	Gastroesophageal reflux disease
GI	Gastrointestinal
GLIM	Global Leadership Initiative on Malnutrition
HAQ-DI	Heath assessment questionnaire disability index
IADL	Instrumental activities of daily living
IL-6	Interleukin-6
ILD	Interstitial lung disease
lSSc	Limited cutaneous Systemic Sclerosis
MedDiet	Mediterranean diet
MMF	Mycophenolate mofetil
mRSS	Modified Rodnan Skin Score
MUST	Malnutrition Universal Screening Tool
ONS	Oral nutrition supplements
PCI	Pneumatosis cystoides intestinalis
PhA	Phase angle
QoL	Quality of life
RDW	Red-cell-distribution-width
SARC-F	Strength, assistance with walking, rise from a chair, climb stairs, and falls
SARC-F + EBM	Strength, assistance with walking, rise from a chair, climb stairs, and falls adjusted for age and body mass
SF-36	36-item short form health survey
SGA	Subjective Global Assessment
SIBO	Small intestinal bacterial overgrowth
SS	Sjögren’s syndrome
SSc	Systemic sclerosis
SSc-ILD	SSc-associated interstitial lung disease
TNF-α	TNF-α: Tumor-necrosis factor-α
UCLA SCTC GIT 2.0	UCLA Scleroderma Clinical Trials Consortium Gastrointestinal Tract 2.0
VAS	Visual analogue scale
